# Inflammation Meets Metabolism: Roles for the Receptor for Advanced Glycation End Products Axis in Cardiovascular Disease

**DOI:** 10.20900/immunometab20210024

**Published:** 2021-06-02

**Authors:** Laura Senatus, Michael MacLean, Lakshmi Arivazhagan, Lander Egaña-Gorroño, Raquel López-Díez, Michaele B. Manigrasso, Henry H. Ruiz, Carolina Vasquez, Robin Wilson, Alexander Shekhtman, Paul F. Gugger, Ravichandran Ramasamy, Ann Marie Schmidt

**Affiliations:** 1 Diabetes Research Program, Department of Medicine, New York University Grossman School of Medicine, New York, NY 10016, USA; 2 The State University of New York at Albany, Albany, NY 12222, USA

**Keywords:** receptor for advanced glycation end products (RAGE), sugar metabolism, cholesterol metabolism, interferon signaling, diabetes, immune cell, monocyte/macrophage, dendritic cell

## Abstract

Fundamental modulation of energy metabolism in immune cells is increasingly being recognized for the ability to impart important changes in cellular properties. In homeostasis, cells of the innate immune system, such as monocytes, macrophages and dendritic cells (DCs), are enabled to respond rapidly to various forms of acute cellular and environmental stress, such as pathogens. In chronic stress milieus, these cells may undergo a re-programming, thereby triggering processes that may instigate tissue damage and failure of resolution. In settings of metabolic dysfunction, moieties such as excess sugars (glucose, fructose and sucrose) accumulate in the tissues and may form advanced glycation end products (AGEs), which are signaling ligands for the receptor for advanced glycation end products (RAGE). In addition, cellular accumulation of cholesterol species such as that occurring upon macrophage engulfment of dead/dying cells, presents these cells with a major challenge to metabolize/efflux excess cholesterol. RAGE contributes to reduced expression and activities of molecules mediating cholesterol efflux. This Review chronicles examples of the roles that sugars and cholesterol, via RAGE, play in immune cells in instigation of maladaptive cellular signaling and the mediation of chronic cellular stress. At this time, emerging roles for the ligand-RAGE axis in metabolism-mediated modulation of inflammatory signaling in immune cells are being unearthed and add to the growing body of factors underlying pathological immunometabolism.

## INTRODUCTION

The Receptor for Advanced Glycation End Products (RAGE) was born from the quest to discern how sugar (glucose)-modified molecules contribute to the pathogenesis of diabetic complications. In first experiments in cultured endothelial cells testing D-glucose-modified albumin (AGE-albumin), as well as other backbone proteins, it was discovered that the AGE-modified proteins, but not the backbone (non-AGE’d proteins), bound to a member of the immunoglobulin superfamily, RAGE [1]. From this discovery, the simultaneous expansion of the biology of AGEs and that of RAGE highlighted novel roles for AGEs beyond diabetes. Furthermore, in-depth study of RAGE uncovered its role as a multi-ligand receptor. These discoveries led to the identification of roles for RAGE in immunometabolism, with implications for disorders such as atherosclerosis, cardiovascular disease and obesity. These introductory sections, to follow, briefly summarize the ligands of RAGE; the body of the Review focuses on examples of settings in which RAGE has been shown to modulate immunometabolic functions.

### AGEs

AGEs are a heterogeneous group of structures that include both fluorescent and non-fluorescent forms [2,3]. Although AGEs have traditionally been measured by biochemical methods, more recently, “skin autofluorescence” has been proposed as a means to estimate AGE content through non-invasive technology [4,5]. Beyond innate in vivo production of AGEs driven by multiple factors, including high levels of blood glucose, food/oral-derived AGEs are considered to be an exogenous source of these species; upon uptake through the gastrointestinal tract, up to 10–30% may become part of a subject’s total AGE burden [6–8]. The balance of the orally-derived AGEs may reach the colon, in which case they might interact with the bacterial microbiota [9]. Although more work needs to be done in this area, studies in human subjects have suggested the possibility that dietary restriction of AGEs might beneficially affect the composition of the gut microbiome and reduce metabolic dysfunction [10,11].

In addition to roles for AGEs in diabetes and diabetic complications, more recent evidence has linked AGEs such as carboxymethyllysine (CML)-AGE to obesity. For example, in the PANACEA study (Physical Activity, Nutrition, Alcohol, Cessation of smoking, Eating out of home in relation to Anthropometry), a sub-cohort of EPIC study (European Prospective Investigation into Cancer and Nutrition), specific common AGEs (CML, carboxyethyllysine (CEL), and *N*^δ^-(5-hydro-5-methyl-4-imidazolon-2-yl)-ornithine (MG-H1) were measured in about 200 foods and a number of factors, particularly body weight, were tracked. It was reported that in European adults, over five years, higher levels of dietary AGEs were associated with marginally greater weight gain [12]. In distinct studies, adipose tissue depots retrieved from obese vs lean subjects displayed higher levels of CML-AGEs, in parallel with higher expression of RAGE [13]. It was reported that in parallel with higher adipose tissue CML-AGE levels, plasma of the obese vs lean subjects contained lower levels of the CML-AGEs; the authors demonstrated that in obesity, higher RAGE expression in the fat tissue “trapped” the CML-AGEs. Furthermore, lower levels of CML-AGE in plasma correlated with higher degrees of insulin resistance. It was notable that these overall conclusions were strengthened by experiments in mice devoid of *Ager* (*Ager* is the gene encoding RAGE) in that study [13].

Although AGEs were the first of the RAGE ligands to be discovered, studies from multiple laboratories have illustrated that RAGE is a multi-ligand receptor. Although the implications of AGE-RAGE interaction in immunometabolism are becoming increasingly apparent, experiments have elucidated that the non-AGE ligands of RAGE also play roles in immune and metabolic pathologies.

### Non-AGE Ligands of RAGE

In addition to AGEs, RAGE is a receptor for multiple members of the S100/calgranulin family; although the first S100s to be identified as RAGE ligands were S100A12 and S100B, a number of other members of this family have been shown to bind and signal through RAGE [14,15]. In addition, high-mobility group box 1 (HMGB1), lysophosphatidic acid (LPA) and oligomeric forms of amyloid beta peptide (Aβ) and islet amyloid polypeptide (IAPP) are also RAGE ligands [16–19]. Among other identified ligands of RAGE are MAC-1, C1q and phosphatidylserine (PS) [20–22].

The RAGE extracellular domains consist of one Variable (V)-type immunoglobulin (Ig) domain followed by two constant (C)-type Ig domains (C1 and C2); although V or VC1 binds to many of the ligand families, the binding sites on the V-domain are heterogeneous and are spatially distinct. In addition, it is established that some of the RAGE ligands may bind solely at the extracellular C1 and C2-type Ig domains [15,23–25]. On account of this diversity of ligand binding to discrete sites on the extracellular domains and that the story of RAGE pathobiological roles in chronic disease is not limited to “one ligand to one disease”, it is plausible that putative therapeutic agents targeting discrete regions on these extracellular domains may display limited efficacy if they do not capture or sequester the biological effects of key mediating ligands in each disease setting.

As noted above, this review will present examples of RAGE roles in immunometabolic perturbations through the lenses of sugars and cholesterol, as key examples by which such metabolic species intersect with the immune system through RAGE.

### GLUCOSE, FRUCTOSE AND SUCROSE: INTERFACING WITH THE RAGE AXIS

**Figure 1.**
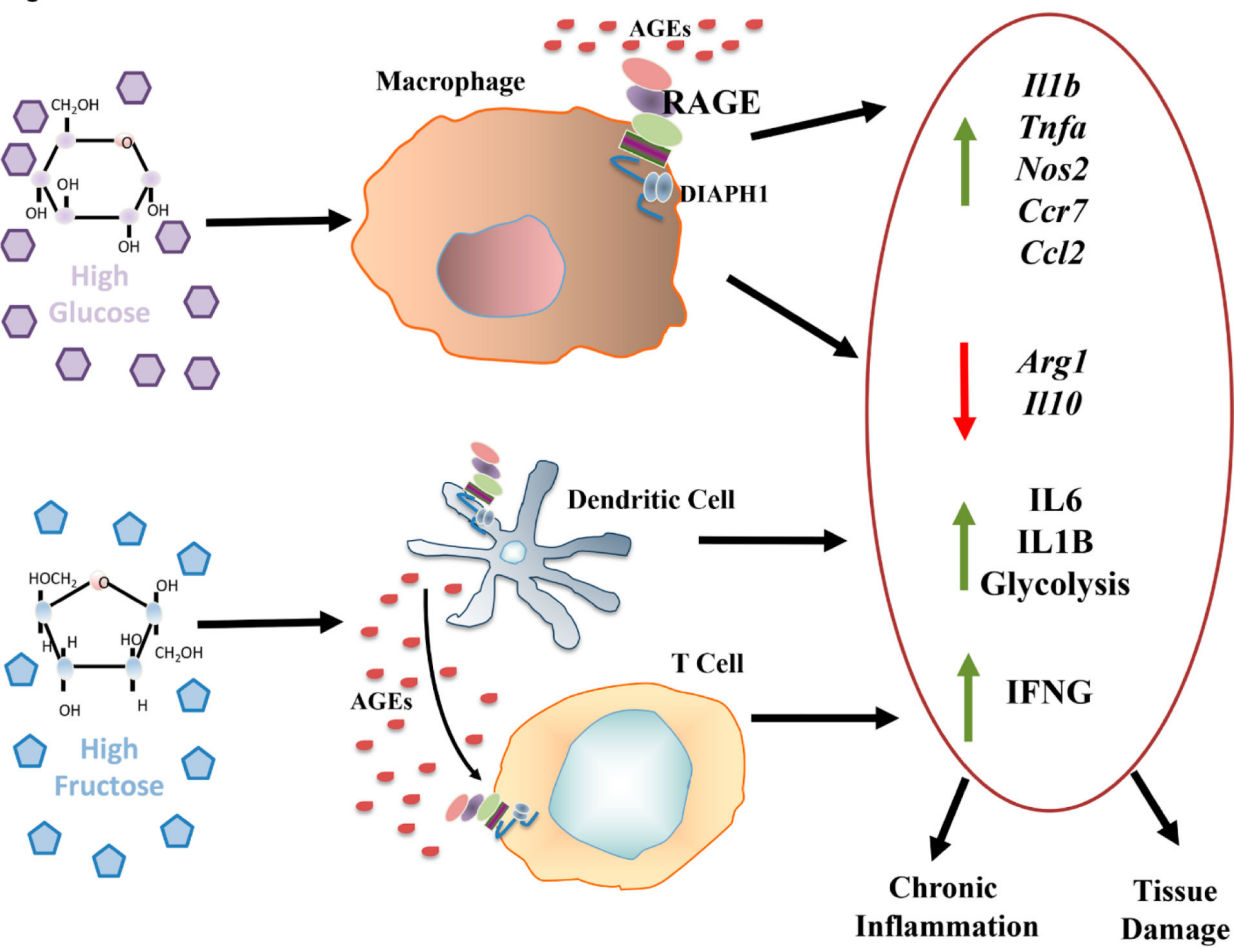
Effect of glucose and fructose on inflammation in murine bone marrow derived macrophages and human dendritic cells and T cells. The sugars glucose and fructose exert significant effects on immune cells. In murine bone marrow-derived macrophages (top), incubation with diabetes-relevant levels of d-glucose (25 mM) vs non-diabetes relevant levels of d-glucose (5 mM) resulted in upregulation of the indicated pro-inflammatory genes, in parallel with downregulation of resolution-provoking genes; these processes were traced to RAGE [26]. In other studies, human peripheral blood-derived DCs incubated with high levels of d-fructose but not d-glucose, increased production of IL1B and IL6 (bottom). Co-incubation of d-fructose-treated DCs with T cells increased T cell production of IFNG. These experiments traced the effects of d-fructose-derived inflammation to RAGE [27].

A common means to test the effects of d-glucose or other sugars on gene expression properties in immune cells, such as macrophages or dendritic cells (DCs), is the exposure of these cells to diabetes-relevant levels of d-glucose (15–25 mM d-glucose) vs homeostatic, non-diabetic levels of d-glucose (5 mM). To study the effects of RAGE on the response to varied glucose conditions, murine bone marrow derived macrophages (BMDMs) were isolated and incubated with diabetes-relevant high levels of glucose (25 mM d-glucose) or euglycemic levels (5 mM). Incubation of BMDMs exerted significant effects on expression of inflammatory mediators; compared to d-glucose (5 mM), treatment of these cells with high glucose resulted in significant upregulation of mRNA transcripts encoding *Il1b, Tnf, Nos2* and *Ccr7* in wild-type cells; these effects were significantly mitigated in BMDMs retrieved from *Ager* null mice [26]. In contrast, high glucose resulted in downregulation of *Egr1* and *Ccl2* in wild-type but not *Ager* null BMDMs. Whereas high glucose downregulated mRNA transcripts for *Arg1* and *Il10* in wild-type BMDMs, these effects were significantly attenuated by deletion of BMDM *Ager* and, in fact, in high glucose conditions, BMDMs devoid of *Ager* displayed even higher levels of these two key mediators linked to anti-inflammatory/tissue repair processes [26] (Figure 1). These findings indicate the pleiotropy of RAGE-dependent actions driven by glucose in immune cells such as macrophages, derived from the bone marrow. Whereas expression of RAGE results in upregulation of certain “pro-inflammatory” mediators, others, such as *Ccl2,* were downregulated by RAGE in BMDMs. Although these studies did not probe how direct metabolic effects contributed to or were consequences of RAGE actions by glucose, others studies have addressed the roles of distinct sugars in immune cells, such as DCs.

In in vitro studies, incubation of human peripheral blood-derived DCs with high levels of d-fructose, but not d-glucose (15 mM), resulted in production of IL6 and IL1B [27]. When T cells were incubated with the d-fructose-treated DCs, production of Interferon (IFN)-gamma (G) (IFNG) was enhanced. The underlying mechanisms were traced to high fructose-mediated AGE generation, which, via RAGE, mediated these effects. In the acute setting (24 h), these effects were not dependent on metabolic changes as there was no evidence of increased glycolysis [27]. However, when DCs were chronically exposed to high fructose (72 h), Seahorse analyzer studies revealed a shift towards glycolysis that was not observed when the cells were treated with glucose. When the authors compared AGE formation and its inflammatory potential driven by glucose vs fructose, their studies suggested that fructose-derived AGEs, via RAGE, were more inflammatory than those stimulated by high glucose [27]. Collectively, these findings implicated fructose-derived AGEs, via RAGE, in upregulation of proinflammatory pathways in human DCs (Figure 1).

The dietary effects of distinct sugars, sucrose and starch (the latter, polymeric carbohydrate consisting of numerous glucose units joined by glycosidic bonds (polymers)), were tested in vivo for their potential effects on hypothalamic inflammation. Microglia, the resident “macrophages” of the central nervous system, play key roles in modulation of properties in surrounding cells in the environment, such as neurons and astrocytes [28]. To test the potential distinct effects of fats vs sugars on hypothalamic inflammation, mice were fed various diets as follows: Diet 1: normal chow (high carbohydrate (64.3%) (wheat and corn), low fat 16.7%); Diet 2: high carbohydrate/high fat (25.5% [sucrose], 58% fat); Diet 3: (high carbohydrate/high fat (19.4% [starch], 61.9% fat); Diet 4: low carbohydrate/high fat (2.2% carbohydrate, 78.7% fat); and Diet 5: low carbohydrate/high fat (1.7% carbohydrate, 92.8% fat). Among the numerous endpoints studied, the authors concluded that only diets high in the carbohydrates (sucrose or starch), but not high fat alone, mediated microglial reactivity and they showed that these effects were due to diet-driven generation of AGEs, acting via RAGE [29]. In parallel, examination of metabolic factors in the mice devoid of *Ager* and fed the high carbohydrate-containing diet revealed that obesity was reduced, as was glucose intolerance, suggesting that AGEs, at least in part via RAGE, mediated these pathological factors in the hypothalami of the mice [29].

### LIGAND-RAGE AXIS: CLEARANCE OF DEAD CELLS, AUTOPHAGY AND MITOPHAGY

Roles for the ligand-RAGE axis in the clearance of dead cells has been considered, as the underlying mechanisms and consequences directly impact metabolic pathways. Examples of such findings in the context of the RAGE axis continue to accrue. The clearance of apoptotic cells, or efferocytosis, is a multi-step process triggered by caspase-dependent apoptosis. This process is essential for the removal of dead/dying cells, both in development and in the maintenance of tissue homeostasis in the adult state, particularly upon imposition of perturbations in which the accumulation of dead/dying cells and their elaborated potentially toxic mediators are maladaptive in the cellular milieu [30,31]. Among the numerous “eat-me” signals on dead/dying cells is the cell surface expression of phosphatidylserine (PS), which was shown to interact with RAGE [22,32,33]. Mice devoid of *Ager* demonstrated a reduced ability to engulf apoptotic neutrophils and thymocytes [33]. In addition to experiments testing genetic deletion of *Ager*, other studies used sRAGE or anti-RAGE antibodies to show decreased phagocytosis of apoptotic cells and in gain-of-function experiments, using overexpression strategies in human embryonic kidney cells (HEK cells), an increased ability to engulf apoptotic cells was illustrated [33]. The specific mechanism was traced to binding of RAGE to PS by use of a solid-state ELISA and an adhesion assay.

Others demonstrated analogous findings; surface plasmon resonance (SPR) was used to show that sRAGE bound to PS (*K*_D_ = 563 nM) [22]. In addition, fluorescence resonance energy transfer (FRET) also confirmed the interaction. In vivo studies were also performed in which lung injury was induced by lipopolysaccharide (LPS); in the *Ager* null mice, there were increased numbers of apoptotic neutrophils in the bronchoalveolar lavage fluid (BAL), suggesting reduced clearance in the absence of RAGE [22]. Those authors also proposed that these findings present interesting insights into how the balance between circulating sRAGE levels and the membrane RAGE (mRAGE) might be crucial in determining the overall effects of RAGE in the clearance of apoptotic cells. As it is well established that levels of sRAGE may vary in human subjects depending on presence or absence of diseases in which RAGE ligands accumulate [34–37], such findings may be important in determining the potential (or not) for the clearance of apoptotic cells, which, themselves, may augment inflammatory responses. Importantly, in addition to RAGE, numerous receptors that recognize PS have been identified [38]. Certainly, such versatility provides for flexibility in discrete organs and, likely, in the setting of distinct perturbants and triggers of severe cellular stress and apoptosis. If and how RAGE may be called upon for roles in these processes remains to be fully elucidated.

Increasing evidence has provided insight into the metabolic reprogramming of innate immune cells, including myeloid cells, in processes spanning glycolysis and oxidative phosphorylation [39]. In microglia, the ligand-RAGE axis has also been linked to mitophagy in responses to chronic stress. In a study by Zhang and colleagues [40], chronic stress induced by intermittent electric foot shocks and noise stress, which was administered two hours/day for 15 consecutive days, resulted in increased cytoplasmic translocations of RAGE ligand HMGB1 and upregulation of RAGE. In the rostral ventrolateral medulla (RVLM), mitochondria and oxidative stress, with consequent proinflammatory polarization of microglia, were reduced in the RVLM of stressed mice devoid of microglia *Ager*. HMGB1-RAGE impaired the late stages of mitophagy flux in these cells, which was associated with increased neuroinflammation (NF-kB pathway) and increased blood pressure in the stressed mice; these processes were relieved by microglia *Ager* deletion or by delivery of siRNAs to reduce expression of *Hmgb1* in the RVLM [40].

Mitophagy is thought to be intricately related to the inflammasome [41], which is known to regulate a distinct form of cell death, known as pyroptosis, which has been studied in the context of RAGE [42]. Pyroptosis is a form of programmed lytic death in immune cells consequent to infection [43]. Once triggered, the purpose of pyroptosis is to eliminate the infected cells, which, in turn, leads to initiation of inflammatory responses [44]. As in other forms of acute responses to stress, however, overactivation of these mechanisms may damage the host. The canonical inflammasome pathway mediates these responses, leading to maturation and activation of IL1beta and IL18 [43,44]. In a murine model of sepsis, RAGE ligand HMGB1 derived from neutrophil extracellular traps (NETs), acting via RAGE and dynamin-dependent signaling, and the downstream molecular pathways, drove macrophage pyroptosis through cathepsin B (CatB) release from ruptured lysosomes, followed by the formation of the pyroptosome and caspase-1 activation [45].

In the case of phagocytes and the engulfment of dead cells, a large burden of metabolic species is immediately presented to the cell. In a recent review, Doran and colleagues identified three examples of mechanisms by which the surge in lipids might be handled by the engulfing phagocyte [46]. First, the free cholesterol may be esterified by acyl-CoA cholesterol acyltransferase (ACAT) into cholesterol esters; consequent to this step, the species may be sequestered in neutral lipid droplets or effluxed from the cell, the latter in an effort to reduce the intracellular accumulation. Cholesterol efflux transporters, such as ATP-binding cassette transporter A1 (ABCA1) may be recruited for this effort. Second, the fatty acids derived from the apoptotic cells may undergo fatty acid oxidation via the mitochondria; this results in the expression of IL10 (resolution pathway). Third, the binding of the apoptotic cells to PS-expressing efferocyte receptors may lead to the upregulation of glucose transporter 1 (GLUT1 or SLC2A1) and serum/glucocorticoid regulated kinase 1 (SGK1), which is required for GLUT1 transport to the plasma membrane [46], thereby regulating glucose metabolism. The latter findings implicate the process of glycolysis in the response to acquired metabolite loads upon the engulfment of dead/dying cells. In this context, glycolysis in macrophages has been suggested to link to pro-inflammatory mechanisms [47–49]. The specific effects of RAGE on glycolysis in macrophages have yet to be fully explored; rather, a number of studies have suggested that the products of glycolysis may generate RAGE ligand methylglyoxal-modified species [50,51]. A number of studies have linked RAGE to cholesterol metabolism and will be considered in the sections to follow.

### RAGE AND CHOLESTEROL METABOLISM

RAGE has been implicated in downregulation of cholesterol transporters. Such effects, if not accompanied by opposing processes implicated in cholesterol metabolism, would have the effect of increasing cholesterol accumulation in macrophages [52–54]. These questions in the context of RAGE have been examined in a number of research studies, as detailed below.

In human macrophages, the effects of exposure to AGE-BSA were detected using microarray analyses followed by PCR studies. In those cells, treatment with AGE-BSA resulted in a reduction in the mRNA of the ATP-binding cassette transporter G1 (*ABCG1*), but no effects on the transporter *ABCA1* were shown [55]. Treatment with anti-RAGE antibodies reduced the downregulation of *ABCG1,* thereby implicating RAGE as a mediator of these effects. In in vitro studies to determine the putative effects on cholesterol efflux to high density lipoprotein (HDL) or Apolipoprotein A1 (ApoA1), it was shown that AGE-BSA reduced cholesterol efflux to HDL but not to ApoA1. Although the regulation of ABCG1 (and ABCA1) has been extensively linked to the Liver X Receptors (LXRs) [56], AGE-BSA did not reduce the mRNA levels of the genes linked to LXRs and had no effect on the binding of nuclear proteins to the LXR response element when compared with treatment with BSA (non-glycated) [56].

A distinct study examined the effect of a different RAGE ligand, S100B, on expression of cholesterol transporters using human THP1 monocyte/macrophage-like cells and human monocytes [57]. In those studies, the authors showed that treatment of THP1 cells with S100B resulted in 2–3-fold reduction in *ABCA1* and *ABCG1* mRNA and that by Western blotting, the levels of ABCA1 protein were also significantly attenuated by S100B. These effects of S100B-mediated suppression of these transporters was attenuated by anti-RAGE antibodies. It was shown that the suppressive effects of S100B were reversed by treatment with ligands of the LXR [57]. Furthermore, the authors isolated peripheral blood mononuclear cells (PBMCs) from diabetic patients and reported that the mRNA levels of *ABCA1* and *ABCG1* were reduced by about 3-fold in the cells from type 1 and type 2 diabetic patients vs non-diabetic control subjects [57]. In terms of molecular regulation by S100B, although the RAGE ligand had no effect on *ABCA1* mRNA stability, it was shown that the effects of S100B on the ABCA1 transporter were dependent on LXR [57].

As implied by the studies discussed immediately above, in addition to reduced levels of the cholesterol transporters ABCA1 and ABCG1 in diabetes, it has been reported that serum cholesterol efflux capacity and reverse cholesterol transport (RCT), the functional indices of the biological effects of these transporters, are reduced in diabetes [58,59]. These findings are suggested to have pathological relevance, as inferred from the documented inverse relationship between cholesterol efflux capacity and carotid-intima thickness, a well-established surrogate marker of atherosclerosis [60,61]. Daffu and colleagues tested the hypothesis that RAGE contributed to these processes in diabetes. In macrophages grown in diabetes-relevant vs euglycemic levels of d-glucose, macrophage cholesterol efflux to ApoA1 and HDL were both reduced and, in vivo, RCT to plasma, liver and feces was significantly reduced in diabetic macrophages, at least in part via RAGE [62]. In vitro, RAGE ligands suppressed macrophage levels of *ABCG1* and *ABCA1*; interestingly, these processes were independent of LXR-dependent mechanisms, but were dependent on peroxisome proliferator-activated receptor-γ (PPARG)-responsive promoter elements [62]. To determine if RAGE contributed to downregulation of these cholesterol transporters in diabetic macrophages in murine atherosclerotic plaques, laser capture microdissection of CD68-expressing macrophages was performed on atherosclerotic plaques of diabetic mice devoid of *Ldlr* expressing or devoid of both the *Ldlr* and *Ager.* In this setting, macrophages devoid of *Ager* displayed higher levels of *Abca1*, *Abcg1* and *Pparg* mRNA transcripts vs those macrophages from diabetic mice expressing *Ager*; these effects of deletion of *Ager* were independent of glycemia [62]. Hence, those studies provided insight into roles for RAGE in diabetes-associated suppression of macrophage cholesterol efflux and RCT.

These studies were recently extended to the study of in vivo-derived AGEs. Albumin was isolated from patients with type 1 and type 2 diabetes (T1D and T2D, respectively) and from non-diabetic control subjects. Macrophages were retrieved from *Ager* null and wild-type mice, or human THP1 cells were subjected to treatment with silencing RNAs to reduce the expression of *AGER* [63]. Compared to wild-type murine macrophages and control-treated THP1 cells, cholesterol efflux was reduced in response to albumin from T1D or T2D patients vs control albumin [63]; in parallel with increased intracellular lipid content. These effects of the human diabetic albumin were reduced in macrophages lacking *Ager;* in that setting, cholesterol efflux and lipid staining were higher and lower, respectively, when compared to the control RAGE-expressing cells [63]. These latter studies are important as they used in vivo-isolated vs in vitro-prepared AGEs; nevertheless, they indicate that the in vitro-prepared material behaves analogously to that of AGEs obtained directly from diabetic subjects.

In a distinct study, the effects of AGEs on macrophage lipid content and underlying mechanisms were examined. It had been previously shown that the AGE-RAGE axis was implicated in the accumulation of lipid droplets accumulation in the aortas of high fat-fed diabetic Goto Kakisaki (GK) rats [64]. As it was also previously reported that AGEs promote foam cell formation [65,66], the role of AGE-RAGE interaction in cholesterol uptake, synthesis and efflux of cholesterol in macrophages was addressed in distinct studies. To comprehensively test this pathway, THP1 cells differentiated into macrophages were treated with AGEs alone or in the presence of modified forms of LDL [67]. The authors reported that AGEs plus modified LDL increased lipid accumulation and cholesterol esters in macrophages, and decreased HDL-mediated efflux of cholesterol through regulation of CD36, scavenger receptors A1, HMG-CoA-reductase, ACAT1 and ABCG1; processes which were reversed in the presence of anti-RAGE antibodies [67].

Collectively, these studies highlight roles for RAGE in regulation of macrophage cholesterol content; processes which appear to be linked to increased uptake and esterification and to downregulation of molecules that are important for efflux of cholesterol from these cells.

Together, these changes in intracellular cholesterol content, particularly in diabetes, may exert significant impact on inflammatory and pro-atherogenic mechanisms.

### RAGE AND ATHEROSCLEROSIS: SUGARS & LIPIDS

Among the complications of diabetes is the development of accelerated atherosclerosis and cardiovascular complications; evidence from long-term clinical studies in T1D and T2D suggest that reduction in glucose levels contributes to reduction of cardiovascular complications [68,69]. However, the protection imbued by rigorous control of hyperglycemia, especially in T2D is not complete and, in fact, may be harmful (due in part to hypoglycemic episodes) [70]. In T1D, after a period of approximately 15 years, the beneficial effects of strict control of hyperglycemia may begin to wane, and usher in a next phase of “metabolic amnesia” [71]. These considerations suggest that while high levels of glucose are key contributing factors, there remain distinct contributors that affect long-term cardiovascular complications.

Studies in human atherosclerosis have illustrated that diabetes is associated with higher expression of lesional RAGE, higher macrophage content and evidence of increased inflammatory and oxidative stress [72,73]. Experiments from multiple groups have suggested that diabetes-accelerated atherosclerosis in mouse models may be mitigated by deletion of *Ager* (globally or in bone marrow-derived cells) [74–76] or by pharmacological agents directed to RAGE ligands and RAGE, such as sRAGE and anti-RAGE antibodies [77–79]. To follow in the sections below are examples of studies linking RAGE, sugars and lipids to immunometabolic consequences in atherosclerosis.

As discussed above, diabetes accelerates atherosclerosis in human subjects and in animal models. Using RAGE-directed antibodies for imaging, prominent RAGE expression was localized to the diabetic atherosclerotic plaques in mice and in pigs [80,81]. As in the clinical studies in T1D, the question was asked, what happens to RAGE expression and atherosclerosis in diabetic mice devoid of *Apoe* and subjected to insulin treatment to lower blood glucose? In that study, *Apoe* null mice were rendered T1D-like diabetic with streptozotocin; after 6 weeks of established diabetes, insulin pellets vs vehicle were implanted in the diabetic mice for an additional 15 weeks. In parallel with decreased glucose levels, lesional RAGE expression and lesion size were reduced by treatment with insulin and macrophage content was significantly reduced in the lesions, as measured by immunoreactivity with Mac-3 [82]. Of note, however, cholesterol levels were not reported in that study; hence, the effects of insulin and reduced hyperglycemia might have resulted, at least in part, through reductions in hypercholesterolemia.

In a distinct study, this issue was directly addressed in studies in murine models of atherosclerosis. To mimic transient intermittent hyperglycemia (TIH), a common metabolic feature observed in human subjects, which has been associated with cardiovascular disease [83,84], TIH was induced through repeated injections of glucose through the peritoneal cavity in *Apoe* null mice. Although TIH accelerated atherosclerosis in the glucose-treated mice, there was no effect on plasma cholesterol, thereby de-coupling the effects of glucose vs cholesterol on atherosclerosis [85]. The mechanisms were traced to promotion of myelopoiesis with increased circulating monocytes (especially the Ly6C^HI^ subset), and were linked to the RAGE ligand S100A8/A9) and to RAGE itself using genetically modified mice and bone marrow [85]. In neutrophils, the increased uptake of glucose uptake via GLUT enhanced glycolysis in these cells, thereby triggering the production of RAGE ligand S100A8/A9, which was linked directly to atherosclerosis through myeloid-specific deletion of *Slc2a1* (GLUT1) [85]. Hence, these studies linked glucose, glycolysis, S100A8/A9 and RAGE in a cholesterol-independent manner to acceleration of atherosclerosis in mice devoid of *Apoe.*

Diabetes is also associated with increased vascular calcification; coronary artery calcification has been associated with increased cardiovascular morbidity and mortality [86]. RAGE ligand S100A9 was linked to calcification mechanisms through high glucose treatment of macrophages. When human primary macrophages were cultured in the presence of high glucose conditions, the secretion of RAGE ligand S100A9 and expression of RAGE in those cells was noted to increase [87]. Recombinant S100A9 was employed to directly test its effects on macrophages; S100A9 induced expression of osteogenic factors and the production of extracellular vesicles that contained high calcific potential (on account of high alkaline phosphatase activity); this was prevented by blockade of RAGE or silencing RNAs targeting *S100a9*. In vivo, streptozotocin-induced T1D-like mice devoid of *Apoe* and *S100a9* or diabetic mice treated with siRNAs targeted against *S100a9* encapsulated in lipid nanoparticles displayed decreased atherosclerosis and microcalcifications in the atherosclerotic plaques [87]. Those authors also tested human carotid plaques and showed that the S100A9-RAGE axis associated with osteogenic activity and microcalcification [87]. Collectively, these studies directly linked high glucose exposure in macrophages to induction of pro-calcific mechanisms linked to atherosclerotic plaque microcalcification.

**Figure 2.**
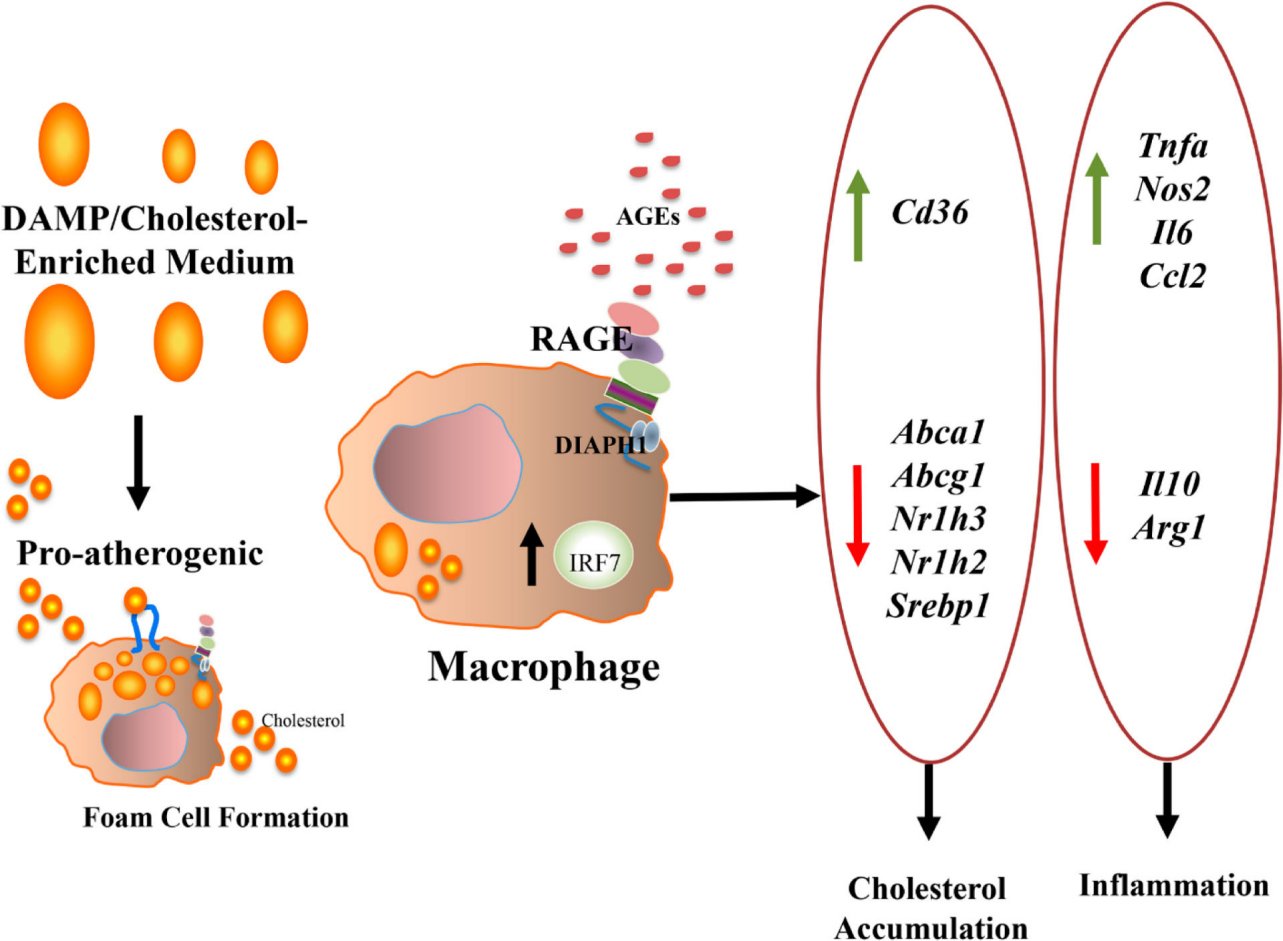
Effect of DAMPs and cholesterol-enriched medium on gene expression in murine bone marrow derived macrophages through IRF7. IRF7 is linked to regulation of cholesterol- and inflammation-modulating genes in macrophages. In murine bone marrow derived macrophages, incubation with DAMP-enriched cholesterol-enriched medium resulted in increased expression of *Abca1, Abcg1*, *Nr1h2* (LXRβ) and *Nr1h3* (LXRα), and decreased expression of *Cd36*; regulation of these factors was reversed by knockdown of *Irf7.* Furthermore, with respect to inflammation, siRNA-knockdown of *Irf7* upregulated *Arg1* and *Il10*, and significantly downregulated *Tnfa*, *Nos2*, *Il6*, and *Ccl2* in bone marrow derived macrophages [92]. In those studies, it was shown that RAGE ligands upregulate *Irf7* in macrophages.

Finally, multiple studies have illustrated that the regression of established atherosclerosis is impaired by diabetes [88–91]. When roles for RAGE were explored in regression of diabetic atherosclerosis, exciting findings emerged that linked diabetes to interferon signaling with consequent effects on macrophage inflammation, glycolysis and cholesterol metabolism, as follows [92]. Regression of atherosclerosis was explored through a model in which transplantation of aortic arches from diabetic, Western diet-fed mice devoid of the *Ldlr* were transplanted into *Ager* null vs wild-type diabetic recipient mice; transfer of the arches into the normolipidemic yet diabetic environment accelerated regression of atherosclerosis. When the arches were transplanted into diabetic *Ager* null mice, regression of atherosclerosis was accelerated, as evidenced by reduced macrophage content, lesion size and Oil Red O content [92]. To dissect underlying mechanisms, RNA-sequencing experiments were performed on macrophages isolated from the donor arches pre-transplant (CD45.2) and from the macrophages retrieved from the regressing plaques in the recipient mice (CD45.1). Analyses revealed that the CD45.1 macrophages retrieved from recipients devoid of *Ager* revealed downregulation of Interferon Regulatory Factor 7 (IRF7) and upregulation of glycolysis pathway compared to the macrophages (CD45.1) from the wild-type mice recipients [92]. Immunohistochemistry studies performed on both human and murine diabetic atherosclerotic plaques colocalized IRF7 and macrophages. Experiments were performed in BMDMs; those studies revealed that RAGE ligands upregulated expression of *Irf7* mRNA, and in BMDMs exposed to a cholesterol-enriched medium, knockdown of *Irf7* modulated expression of genes linked to inflammation and cholesterol metabolism. Specifically, knockdown of *Irf7* increased expression of genes linked to cholesterol efflux (*Abca1* and *Abcg1*); increased expression of *Nr1h2* (LXRβ) and *Nr1h3* (LXRα); and downregulated expression of *Cd36* compared to control siRNAs. In terms of inflammatory pathways, knockdown of *Irf7* upregulated *Arg1* and *Il10*; and significantly downregulated *Tnfa*, *Nos2*, *Il6*, and *Ccl2* [92] (Figure 2). Although the potential effects of RAGE and, possibly, IRF7 on glycolysis in macrophages (based on the analysis of the RNA-sequencing studies) were not directly studied in that work, the overall “glycolysis” pathway was found to be increased in recipient macrophages devoid of *Ager* in the atherosclerosis regression environment. Hence, such findings form the basis of future investigation to explore RAGE/IRF7 links to glycolysis—cause and/or effect?

### RAGE AND OBESITY: EVIDENCE FOR ROLES FOR ENERGY EXPENDITURE AND INSULIN RESISTANCE

Even in the absence of diabetes, obese adipose tissue from human subjects and animal models fed a high fat diet (HFD) displays higher levels of AGE, RAGE and other ligands, such as HMGB1 [93]. Consistent with important roles for RAGE in the response to HFD feeding in mice, it was reported that mice globally devoid of *Ager*, when fed a HFD, displayed less weight gain, in parallel with higher energy expenditure and reduced adipose tissue inflammation (reduced macrophage and reduced F4/80+/CD11C+ cells in epididymal adipose tissue (eWAT)) [93]. In these mice devoid of *Ager*, insulin tolerance testing and hyperinsulinemic euglycemic clamp studies illustrated that the *Ager* null mice were remarkably more insulin sensitive. *Ager* deficiency had no effect on genetic forms of diabetes induced by obesity, such as in animals with disruption of melancortin signaling [93].

In the search for underlying mechanisms, mice bearing adipocyte-specific deletion of *Ager* (brown and white adipocytes) displayed significant protection from HFD feeding and from cold challenge. Energy expenditure was significantly higher in the HFD-fed adipocyte *Ager*-deleted animals vs floxed mice controls (*Adipoq* cre recombinase) [94], in a manner independent of changes in food intake or physical activity. In parallel, insulin and glucose tolerance was improved in the adipocyte-*Ager-*deleted mice and eWAT displayed lower expression of pro-inflammatory genes compared to the wild-type control mice [94]. RAGE-dependent mechanisms were mediated by ligand-RAGE-induced suppression of protein kinase A (PKA)-mediated phosphorylation of targets, hormone-sensitive lipase and p38 mitogen-activated protein kinase, upon β-adrenergic receptor stimulation, which culminated in reduced expression of UCP1 [94].

These investigations regarding the RAGE axis were extended to human subjects with obesity or morbid obesity, in which case subcutaneous and omental adipose tissues (SAT and OAT, respectively) were obtained at the time of bariatric surgery and experiments were performed to determine if there associations between *AGER* mRNA expression and markers of immunometabolic perturbations. In SAT but not OAT, *AGER* mRNA expression significantly and positively correlated with *CD68* mRNA (marker of macrophages) and with *PPARG* [95]. Strikingly, in SAT but not in OAT, only *AGER* expression, but not expression of *CD68* or *PPARG*, was significantly associated with HOMA-IR (homeostatic model assessment for insulin resistance) [95]. In SAT and OAT, no differences in AGE content were identified, thereby indicating that the expression pattern of *AGER* mRNA and associations with immunometabolic markers were independent of AGE ligand levels in these adipose tissue depots [95].

Taken together, in the setting of cardiometabolic disorders, evidence supports that the ligand-RAGE axis impacts metabolism in macrophages and other myeloid cells, at least in part through the effects of glucose and cholesterol species.

## SUMMARY AND PERSPECTIVES

**Figure 3.**
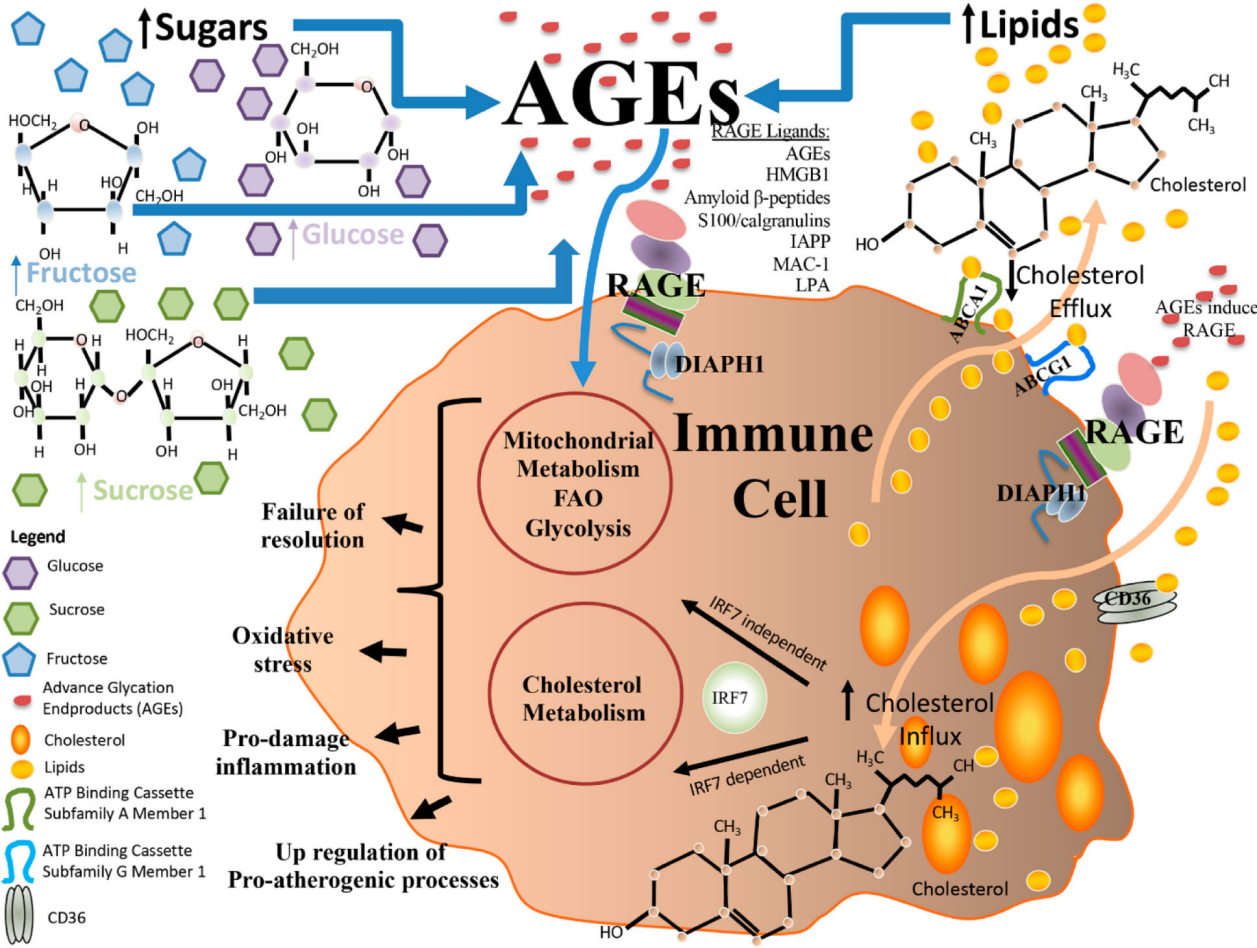
RAGE and Roles in Immunometabolism: an evolving biology in the sphere of sugars, lipids and cholesterol. RAGE plays numerous roles in immunometabolism and research has unveiled that a number distinct biochemical species drive these responses. For example, studies have identified roles for sugars such as sucrose, fructose and glucose in the production of AGEs, which, via RAGE, may exert numerous downstream consequences, such as exacerbation of pro-damage inflammation; failure of resolution of inflammation; oxidative stress and up-regulation of atherogenic processes. Such downstream consequences affect the properties of immune cells, such as macrophages and DCs. In addition, it has been shown that RAGE expression in immune cells, such as macrophages, plays important roles in the handling of cholesterol, such as in cellular uptake, esterification and, as shown in numerous studies, in efflux, at least in part through RAGE ligand-mediated downregulation of cholesterol transporters *Abca1* and *Abcg1*. In this context, the increased level of intracellular lipid species may, particularly in pro-atherogenic or diabetic environments, lead to oxidation of these moieties, thereby further exacerbating cellular stress and dysfunction. In addition, recent insights have uncovered roles for RAGE in regulation of IRF7, which, based on the functions of IRF7, highlights a molecular bridge for these pathways in regulation of RAGE ligand-dependent cholesterol metabolism and inflammation.

Recent insights into immunometabolism have illustrated the integration of the inextricably linked processes regulating inflammatory (such as pro- vs anti-inflammatory/resolution responses) and metabolic pathways (such as glycolysis, amino acid metabolism, oxidative phosphorylation, fatty acid oxidation and mitochondrial processes that generate both ATP and, in certain cases, excess oxygen free radicals) (Figure 3). In the context of the RAGE pathway, evidence supports that numerous types of sugars, glucose, sucrose and fructose, for example, may form advanced species such as AGEs that trigger RAGE activities in vitro and in vivo. In addition, observations from a number of groups support that macrophage RAGE is important for cholesterol metabolism, at least in part through regulation of genes linked to downregulation of cholesterol efflux, such as *Abca1* and *Abcg1*; processes which appear to be independent of the LXRs. Recent findings linked RAGE to IRF7 and regulation of cholesterol transporters in macrophages may be the heretofore unrecognized connecting mechanism and deserves further experimental attention (Figure 3). Collectively, these findings unveil that there are multiple future directions; a few of these are considered below.

RAGE is a Damage Associated Molecular Pattern (DAMP) receptor; DAMPs are recognized as the released products of distressed or damaged cells that themselves may inherently trigger immune responses [96]. Examples of these include AGEs, HMGB1, multiple members of the S100/calgranulin family, amyloid beta peptide and DNA [96]. What is also well-appreciated is that many of these molecules, in addition to their ability to bind to RAGE, may also interact with other DAMP receptors, particularly the toll like receptors (TLRs), such as TLR2, TLR4, and TLR9. TLRs have been associated with integration with interferon signaling pathways; recent discoveries linking RAGE ligands to regulation of *Irf7* in macrophages further strengthen the molecular bridges between these two innate immune signaling effector pathways. Critical to discerning the safety and predicted effectiveness of blockade of RAGE in chronic disease will be its cooperation with or antagonism of TLR pathways. It is predicted that the cellular selection of TLR and/or RAGE is likely to be cell type specific, context specific and, likely, mediated by the distinct stimulating DAMPs.

Earlier work in liver injury suggested that RAGE and TLRs might have distinct functions; compared to wild-type mice undergoing 85% (massive hepatectomy), mice devoid of *Myd88* (downstream signaling effector for some of the TLRs) displayed reduced survival. In contrast, mice devoid of *Ager* displayed higher survival compared to the wild-type or *Myd88* null mice. However, mice devoid of both *Ager* and *Myd88* succumbed rapidly, consistent with the survival patterns experienced by the wild-type or the *Myd88* null mice [97]. Such data suggest that at least in that setting, MYD88 plays protective functions in the response to liver injury, whereas RAGE acts to blunt survival responses. Since the double-null mice behave more similarly to the mice devoid of *Myd88*, it may be intuited that TLRs are fully essential for immediate and innate survival responses in that setting [97]. If and how RAGE vs TLR modulated immune cell metabolism in massive hepatectomy was not addressed in that work, but forms the basis for future investigations.

The recent discovery of links between RAGE and interferon pathways bears implications beyond regulation of macrophage inflammation and cholesterol metabolism. IRF7, in particular, has been described as the master regulator of type 1 interferon responses, which are essential for rapid response to multiple types of viral infections [98]. Like RAGE, IRFs such as IRF7 are complex as well; a recent study showed that IRF7 regulated pro-fibrotic responses through transforming growth factor beta pathways [99]; concepts which might well extend a molecular and metabolic bridge between RAGE and IRF7 to cardiovascular diseases and to diseases of the diabetic kidney, which bear strong links to RAGE signaling [100]. In the context of viral infections, dysregulation of interferon pathways has been suggested as one component of the host maladaptive responses to SARS-CoV-2 infection [101–104]. Given the multiple and diverse DAMP ligands of RAGE that are likely to be produced and released in SARS-CoV-2 infection [105], by immune cells and endogenous lung epithelial cells, it will be very interesting to discover if and how RAGE might contribute to interferon dysregulation in the infected lung.

In this context, a recent study directly tested the effects of glucose and glycolytic metabolism on macrophage properties in SARS-CoV-2 infection [106]. In that work, it was shown that SARS-CoV-2 stimulates the production of mitochondrial reactive oxygen species production in macrophages, which results in the stabilization of hypoxia-inducible factor-1α (HIF-1α) and the promotion of glycolysis [106]. The effects of HIF-1α in monocyte metabolism upon SARS-CoV-2 infection cause direct effects on inhibition of T cell responses with consequent reductions in epithelial cell survival [106]. Hence, future studies might focus on the potential roles for RAGE, glucose, metabolism and altered monocyte and immune responses upon SARS-CoV-2 infection in order to discern if, and by what mechanisms, RAGE might contribute to pathobiological responses to this infection.

Finally, with respect to therapeutic opportunities, it is well-established that the cytoplasmic domain of RAGE is essential for RAGE-dependent signaling [107]. The cytoplasmic domain of RAGE binds to distinct intracellular molecules [108,109]; among the best-characterized of these is the interaction of the RAGE cytoplasmic domain with Diaphanous-1 or DIAPH1, a member of the formin family [110,111] (Figure 3). Formins have particular characteristics that render their potential roles in RAGE signaling quite plausible; DIAPH1 is an effector of RhoGTPase signaling; formins play key roles in the actin cytoskeleton, thereby implicating them in cellular migration; and formins may regulate gene expression, at least in part through serum response factors (SRFs), which have been previously linked to RAGE [112]. Structural biology studies unveiled the precise amino acids in the cytoplasmic tail of RAGE (human, R5/Q6), which were required for binding to the Formin Homology 1 domain (FH1 domain) of DIAPH1 [23,113]. Based on this knowledge, small molecule antagonists of this interaction are now under study and development as putative novel antagonists of RAGE signaling [114]. If and to what degree such modulation might re-set adaptive immunometabolic roles for RAGE in chronic disease settings remains to be dissected. If successful, novel therapeutic agents for disorders of metabolism, such as diabetes, obesity, atherosclerosis and cardiovascular disease, among others, may be introduced for clinical trial assessment.
